# Blowin' in the wind: Dispersal of Glossy Ibis *Plegadis falcinellus* in the West Mediterranean basin

**DOI:** 10.1002/ece3.9756

**Published:** 2023-01-19

**Authors:** Boudjéma Samraoui, Riad Nedjah, Abdennour Boucheker, Abdelhakim Bouzid, Hamed A. El‐Serehy, Farrah Samraoui

**Affiliations:** ^1^ Laboratoire de Conservation des Zones Humides Université 8 Mai 1945 Guelma Guelma Algeria; ^2^ Department of Biology University Badji Mokhtar Annaba Algeria; ^3^ Department of Ecology University 8 mai 1945 Guelma Guelma Algeria; ^4^ Département de Sciences Agronomiques University Kasdi Merbah Ouargla Algeria; ^5^ Department of Zoology, College of Science King Saud University Riyadh Saudi Arabia

**Keywords:** circular statistics, connectivity, finite mixture distribution, GAMLSS, metapopulation, natal dispersal, population dynamics, waterbirds

## Abstract

The movement of organisms is a central process in ecology and evolution, and understanding the selective forces shaping the spatial structure of populations is essential to conservation. Known as a trans‐Saharan migrant capable of long‐distance flights, the Glossy Ibis *Plegadis falcinellus*' dispersal remains poorly known. We started a ringing scheme in 2008, the first of its kind in North Africa, and ringed 1121 fledglings over 10 years, of which 265 (23.6%) were resighted. Circular statistics and finite mixture models of natal dispersal indicated: (1) a strong West/Northwest‐East/Southeast flight orientation; (2) Glossy Ibis colonies from North Africa and Southern Europe (particularly on the Iberian Peninsula) are closely linked through partial exchanges of juvenile and immature birds; (3) unlike birds from Eastern Europe, North African Glossy Ibis disperse to but do not seem to undergo regular round‐trip migration to the Sahel; (4) young adults (>2‐years‐old) have a higher probability of dispersing further than individuals in their first calendar year (<1‐year‐old); and (5) dispersal distance is not influenced by sex or morphometric traits. Together, these results enhance our knowledge of the dispersal and metapopulation dynamics of Glossy Ibis, revealing large‐scale connectivity between the Iberian Peninsula and Algeria, likely driven by the spatial heterogeneity of the landscape in these two regions and the prevailing winds in the Western Mediterranean.

## Graphical abstract

The Glossy Ibis Plegadis falcinellus is known as a trans‐Saharan migrant capable of long‐distance flights. Circular statistics and finite mixture models of natal dispersal indicated: (1) a strong West/Northwest‐East/Southeast flight orientation; (2) Glossy Ibis colonies from North Africa and Southern Europe (particularly on the Iberian Peninsula) are closely linked through exchanges of juvenile and immature birds. The metapopulation dynamics of Glossy Ibis are likely driven by the diversity of wetlands in these two regions and the prevailing winds in the Western Mediterranean.

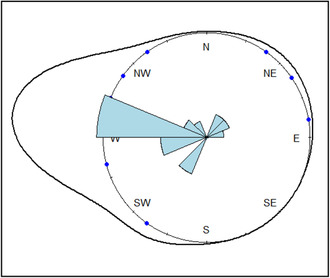

## 1 INTRODUCTION

Migration is an evolutionary adaptation that allows organisms to undertake a directional and synchronized seasonal movement to track down fluctuating or patchy resources or to escape from temporarily adverse conditions (Dingle & Drake, 2007). Another type of animal movement involves dispersal, a key process in ecology and evolution (Clobert et al., 2012). Dispersal—defined as a unidirectional movement of an individual from its natal or breeding site—is also a complex and multidimensional trait influencing community structure, population dynamics, and connectivity (Greenwood & Harvey, 1982; Levin et al., 2003). In particular, natal dispersal—defined as the displacement of individuals from their site of birth as they search for their first breeding site—is believed to play a key role in population dynamics and genetic structure (Ims & Andreassen, 2005). Thus, the study of dispersal behavior is relevant to various biological fields (evolution, population ecology, population genetics, disease ecology, behavior, and conservation biology) (Bauer & Hoye, 2014; Gaines & McClenaghan Jr, 1980; Saastamoinen et al., 2018). Dispersal behavior may be affected by age, sex, body size and time of birth, and there is a consensus that it is not a random process given significant differences in phenotypic characters between dispersers and their philopatric conspecifics (Clarke et al., 1997; Dhondt & Hublé, 1968).

Source population dynamics, such as those relating to population density and dominance status, may also influence the propensity to disperse (Baines et al., 2019). Moreover, environmental and social cues may interact with individual phenotypes to impact dispersal (McCauley, 2010). Thus, although dispersal tends to homogenize neighboring gene pools, dispersers may differ from nondispersers in terms of body size or other phenotypic traits (Camacho et al., 2019; Endriss et al., 2019; Garant et al., 2005). For instance, Fleischer et al. (1984) reported that the smallest female house sparrows (*Passer domesticus*) dispersed the furthest, and Altwegg et al. (2000) found that low‐ranking males in the size hierarchy of broods that hatched early were more inclined to disperse. Thus, factors favoring dispersal appear to vary according to reproductive strategies (Edelman, 2011), potentially accounting for size‐ or sex‐biased dispersal in many species (Fleischer et al., 1984; Forero et al., 2002). Despite extensive research effort being devoted to dispersal (Hamilton & May, 1977; Hansson et al., 2003; Paradis et al., 1998, 2002; Tarwater & Beissinger, 2012), it remains challenging to understand the mechanisms that drive dispersal (Kokko & Lopéz‐Sepulcre, 2006; Møller et al., 2006).

Owing to its considerable dispersal ability, the Glossy Ibis *Plegadis falcinellus* is a widespread waterbird that has managed to colonize both the Old and the New Worlds (Patten & Lasley, 2000; Santoro et al., 2016). This nomadic species is known to alternate periods of stasis with bursts of range expansion, as documented in the middle of the past century when, for reasons that remain unclear, the Glossy Ibis became extinct as a breeding species across the Mediterranean basin (del Hoyo et al., 1992; Snow & Perrins, 1998). This extinction event was accompanied by a progressive decline in the breeding populations of Eastern Europe and the Black and Caspian seas (BirdLife International, 2022; Schogolev, 1996; Zwarts et al., 2009).

However, in sharp contrast to the current downward global biodiversity trend, the Glossy Ibis has staged a remarkable comeback in the Mediterranean region, and other populations are increasing in a number of regions across the world (Patten & Lasley, 2000; Underhill et al., 2016). Likewise, after a century‐long absence from Algeria (Samraoui, Boucheker, et al., 2012), the Glossy Ibis most probably reappeared in its former haunts of Lake Fetzara and Lake Tonga in the 1990s, before then spreading to neighboring sites (Boucheker et al., 2009). Although the Glossy Ibis is at present categorized in the IUCN Red List as “Least Concern” (BirdLife International, 2022), there is a clear need to monitor medium‐ and long‐term population trends and to identify factors that influence population change. For these reasons, this species is now considered one of the most important metapopulation models in the Mediterranean (Santoro et al., 2013).

Most importantly, the key factors driving Mediterranean extirpation of the Glossy Ibis in the recent past have eluded researchers thus far. Similarly, we know little about the evolutionary and ecological processes associated with their dispersal capability and rapid expansion, despite obvious conservation implications (Perkins et al., 2013; Yoder et al., 2010). Indeed, rapid expansion is a key feature of invasive species and a potential response to climate change (Ausden et al., 2019; Santoro et al., 2013; Walther et al., 2009).

This study is part of a larger research program focused on establishing the population dynamics of Algerian waterbirds (Baaloudj et al., 2012; Boucheker et al., 2011; Samraoui et al., 2011), and the influence of nonrandom dispersal on the phenotypic trajectories of West Mediterranean metapopulations. We aimed to determine:

1. whether the dispersal pattern of the Algerian population of Glossy Ibis is randomly distributed, and
2. whether dispersal distance within the West Mediterranean basin is phenotype‐dependent (i.e., if morphological characteristics, sex and/or age influence dispersal propensity).

## 2 METHODS

**FIGURE 1:**
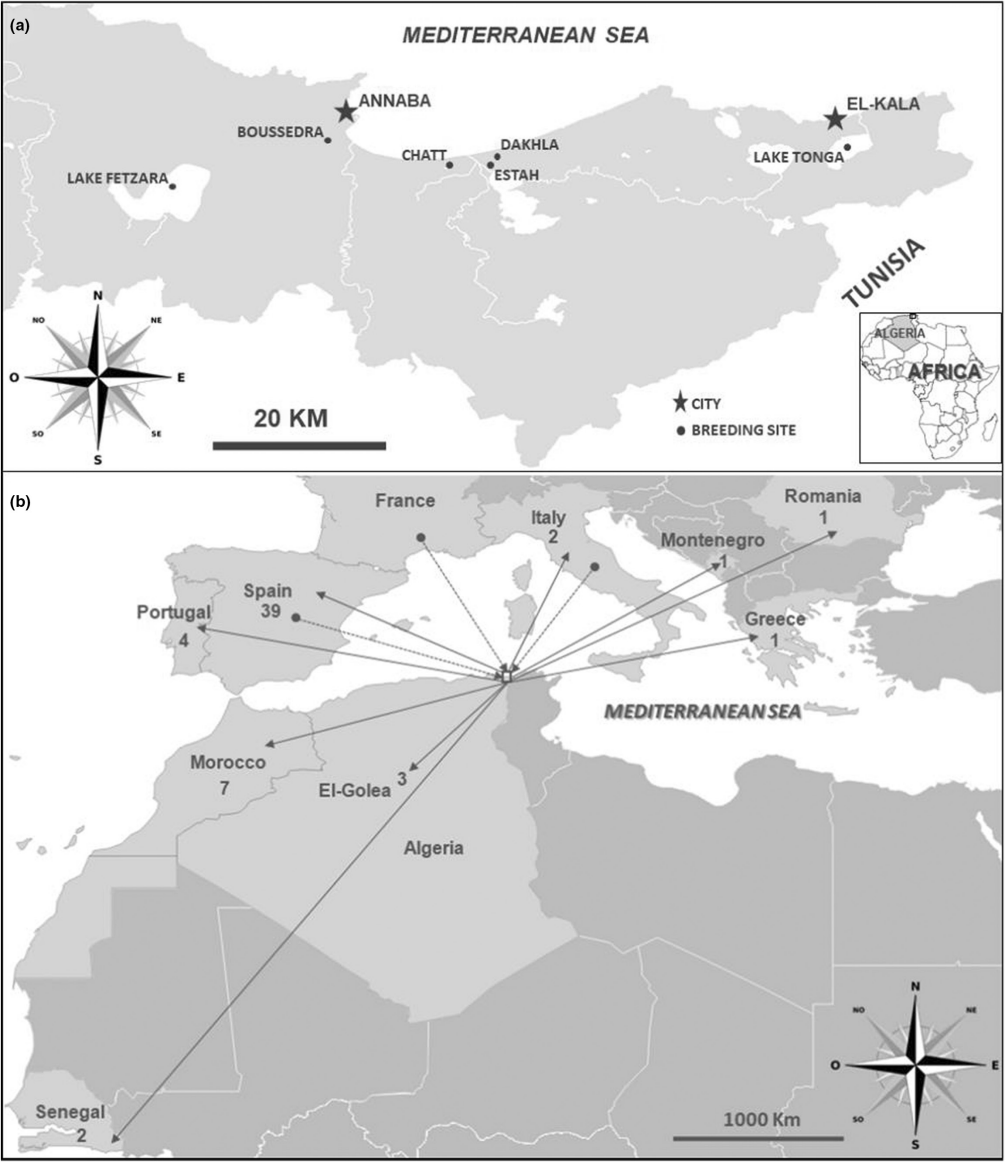
(a) Locations of the breeding colonies in Numidia, northeastern Algeria. (b) Total numbers of Glossy Ibis originating from Numidia and resighted in Europe or West Africa (solid arrows leaving Algeria), as well as birds originating in Europe and resighted in Numidia (dotted arrows).

Ringing of Glossy Ibis was initially carried out in 2008 at Chatt, Algeria (Samraoui, Boucheker, et al., 2012), and was then extended to new colonies (Lake Tonga, Dakhla, Estah, Boussedra and Lake Fetzara) over the following 10 years (Nedjah et al., 2019) (Figure 1a). Each chick 12–16 days old was ringed with a unique engraved PVC band, allowing it to be individually recognized. The rings had a white background with the following specific code: JXX or 0XXX, where X is an alphanumeric character. Head, wing, and tarsus lengths were measured to the nearest mm, and body mass was measured to the nearest gram. A feather was sampled for DNA extraction, and sex determination was performed by means of polymerase chain reaction (PCR) amplification of the *CHD* genes (Griffiths et al., 1998).

Between October 2008 and January 2019, resighting data on Glossy Ibis recorded alive outside their colonies were compiled across major Algerian wetlands and their environs (Samraoui & Samraoui, 2008). The number of resightings represents the number of individual birds observed once or more after fledging. Resightings in Algeria were recorded monthly at various sites and intermittently elsewhere. Records were primarily collected by researchers of the Laboratoire de Conservation des Zones Humides, University of Guelma, and an international network of birdwatchers. Resightings outside Algeria were derived from records of amateur ornithologists and staff of local research and conservation institutions. Resightings in these regions varied according to the undocumented sampling efforts of these professionals and amateurs.

Our dispersal records of Glossy Ibis are best interpreted as circular data that can be analyzed according to directional statistics (Jammalamadaka & SenGupta, 2001). To test for a departure from isotropy (i.e., records evenly distributed across all directions), we used a Rayleigh test, which is particularly suited to unimodal distributions. We also performed an “omnibus” test (Rao's spacing test of uniformity) to test the null hypothesis of uniformity across records (Mardia & Jupp, 1999). To test for uniformity against a unimodal alternative with a specified mean direction, we used a modified version of the Rayleigh test (Agostinelli & Lund, 2013).

We calculated loxodromic distances and dispersal directions from the GPS coordinates of ringing and resighting sites (Imboden & Imboden, 1972). Dispersal distances were modeled using a GAMLSS (Generalized Additive Model for Location, Scale, and Shape) approach, whereby a parametric distribution is fitted to the response variable while the parameters of the distribution are allowed to vary according to the covariates (Rigby & Stasinopoulos, 2005, 2013). To avoid pseudoreplication, we used one record per individual bird, selecting the maximum distance traveled by each individual. Explanatory variables included age class (juveniles—individuals recorded in their first calendar year (1Y) or adults—individuals recorded in their second calendar or subsequent years (2Y+)), sex, and the morphometric variables of fledgling mass, wing length, and tarsus length. Estimates of average dispersal distance and variance were determined using a finite mixture model with no parameters in common with the multimodal data (Everitt & Hand, 1981). First, we compared model fit for a selection of two‐component mixtures of continuous distributions (normal, gamma, inverse Gaussian, Weibull, reverse Gumbel, and logistic distributions), and then selected the best fit using the lowest Akaike information criterion (AIC). Models were validated by inspecting diagnostic plots. We used the packages *circular*, *gamlss*, and *gamlss.mx* available within the software R (R Development Core Team, 2021) to perform all analyses. All means are shown ±1 standard deviation, unless stated otherwise.

## 3 RESULTS

### 3.1 Ringing and resightings

We observed a doubling in the number of Glossy Ibis colonies between 2008 and 2018, increasing from three to six (Table 1). In total, we ringed 1121 individual Glossy Ibis chicks during that period, with 605 resightings of ringed birds over the 11‐year ringing program, corresponding to 265 individuals (23.6% of ringed chicks). Most resightings occurred in Algeria (Table 2), with a mean number of observations for each recorded ring of 2.4 ± 2.9 (*N* = 213 rings) and 1.7 ± 1.5 (*N* = 58 rings) within and outside that country, respectively (Table 3). Numbers of resighted birds varied considerably across Numidia, that is, the northwest region of Algeria: 38.1% (Dakhla); 23.3% (Lake Fetzara); 20.3% (Chatt); 16.5% (Boussedra); 1.1% (Estah); and 0.4% (Lake Tonga).

**TABLE 1:** Annual ringing totals, total numbers of resightings, and resighting data for each Numidian colony from October 2008 to January 2019

Year	Chatt	Dakhla	Fetzara	Tonga	Boussedra	Estah	Total
2008	41	0	26	4	0	0	71
2009	0	58	0	0	0	0	58
2010	96	0	156	0	0	0	252
2011	0	55	0	0	0	0	55
2012	0	103	9	0	0	0	112
2013	74	0	0	0	0	0	74
2014	0	61	0	0	0	0	61
2015	0	66	42	0	0	0	108
2016	9	0	0	0	115	29	153
2017	83	0	0	0	0	0	83
2018	0	72	0	0	0	22	94
Total	303	415	233	4	115	51	1121
Percentage of ringed birds	27.0	37.0	20.8	0.4	10.3	4.6	100
N° resightings	120	192	235	3	51	4	605
N° of birds resighted	54	101	62	1	44	3	265
Percentage of birds resighted	20.3	38.1	23.3	0.4	16.5	1.1	100

*Note*: The overall percentage is based on resightings of individuals anywhere.

**TABLE 2:** Numbers of resightings and Glossy Ibis individuals from Mediterranean countries recorded in Numidia, northeastern Algeria

Origin	Numidia	Spain	Italy	France	Total
Resightings	508	47	27	2	584
Individual birds	213	17	3	1	234

**TABLE 3:** Summary of resighting records for Algerian‐born Glossy Ibis in Numidia and elsewhere

	Numidia	Sahara	Morocco	Senegal	Spain	Portugal	Italy	Montenegro	Romania	Greece	Total
Resightings	508	3	10	2	64	15	3	1	1	1	608
Individual birds	213	3	7	2	39	4	2	1	1	1	273

*Note*: Two birds were observed in two distinct countries (Morocco & Spain and Italy & Montenegro, respectively).

### 3.2 Connectivity between Mediterranean colonies

The Algerian Glossy Ibis population exhibited a high degree of philopatry as the majority of resightings (87.0%) recorded in Numidia were of Algerian‐born birds, with the remaining sightings there attributable to birds hatched in Europe (Table 2). Ten of the immigrant birds were recorded as breeding in Numidia, indicative of significant gene flow between Mediterranean colonies. Although two ringed birds were resighted in West Africa, there were no further observations and so there is no strong evidence for regular movement between this region and Algeria. One bird (JJJ) ringed at Lake Fetzara in 2010 was resighted at El Goléa in the Sahara on March 2012, before returning to Numidia in May later that year, where it has settled ever since.

### 3.3 Dispersal orientation

Dispersing Glossy Ibis from Numidia fanned out primarily across the Mediterranean, but with a strong West/Northwest bias (Figure 1b). The most frequent foreign destination for Algerian‐hatched Glossy Ibis was the Iberian Peninsula (accounting for 71.7% of resighted birds), followed by Morocco (11.7%). There were no resightings or ring recoveries from the Eastern Sahel or Middle East. Notably, the majority of immigrant birds to Algeria (*N* = 17) also came from the Iberian Peninsula. A further three birds came from Italy, and one traveled to Algeria from a French colony.

**FIGURE 2:**
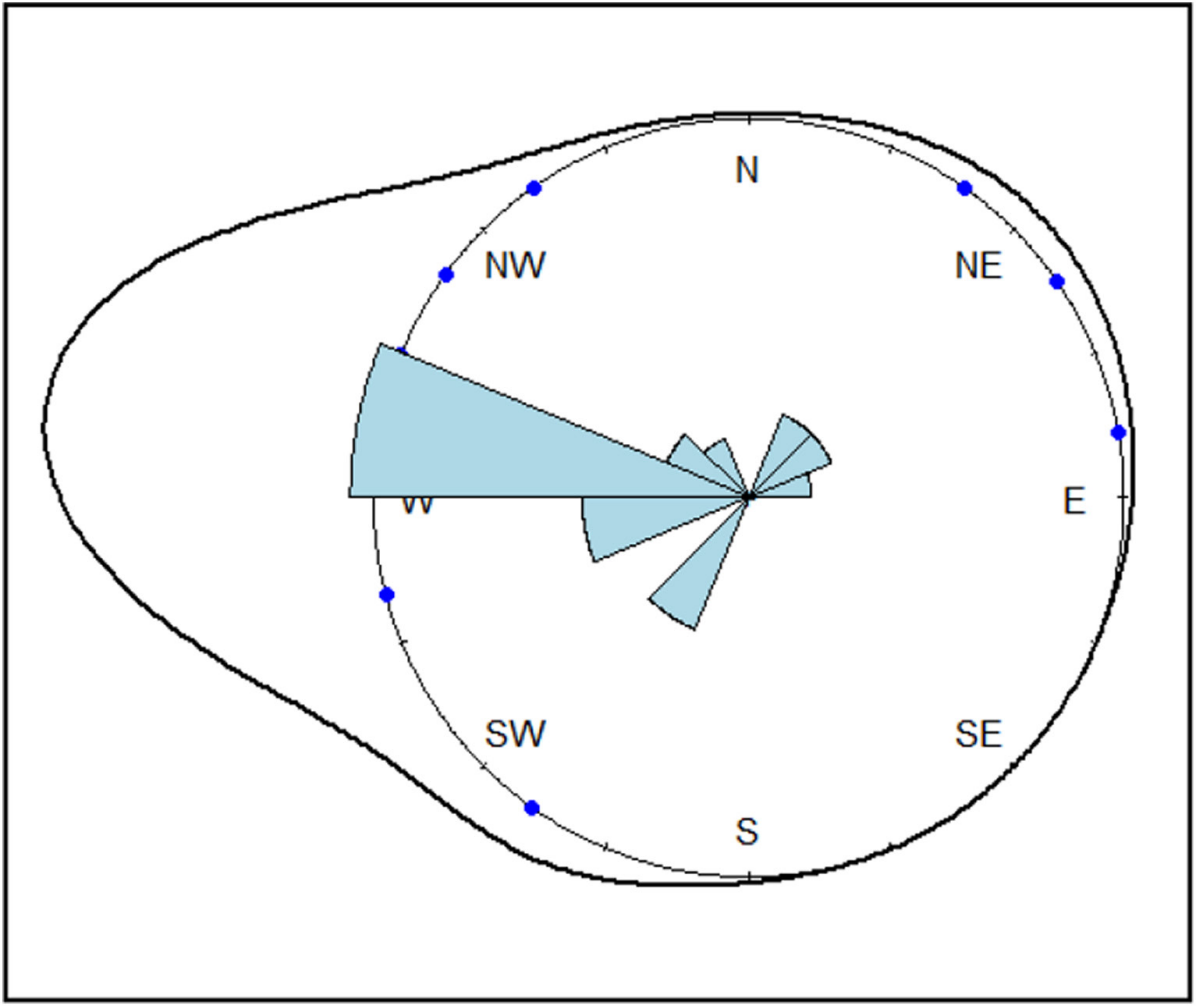
Rose diagram of all records outside of Numidia with kernel density estimates based on the von Mises distribution. An eastern direction was arbitrarily selected as a reference point (Ө = 0).

In Figure 2, we display a rose diagram showing kernel density estimates derived from the von Mises directionality distribution (Mardia & Jupp, 1999). We calculated a sample mean resultant length R of 0.8, representing a measure of the concentration of unimodal circular data, with a sample circular variance of 0.20. The sample mean Ө, reflecting the direction of the mean resultant vector of all records, was 3.03 radians (173.8°), corresponding to a W/NW direction. The Rayleigh test returned a highly significant test statistic of 0.80 (p = 3.16 e‐17), and the Rao's spacing test of uniformity that accounts for potentially multimodal data provided a test statistic of 293.0 (*p* < .001). Based on both test results, we rejected the null hypothesis of isotropy for Glossy Ibis resighting records outside of Algeria. The modified Rayleigh test using a specified mean direction (3.03 radians) also generated a highly significant test statistic of 0.80 (*p* = .0), prompting us to reject the null hypothesis of uniformity in favor of a unimodal distribution with a mean direction of 173.8°.

### 3.4 Dispersal distance

**FIGURE 3:**
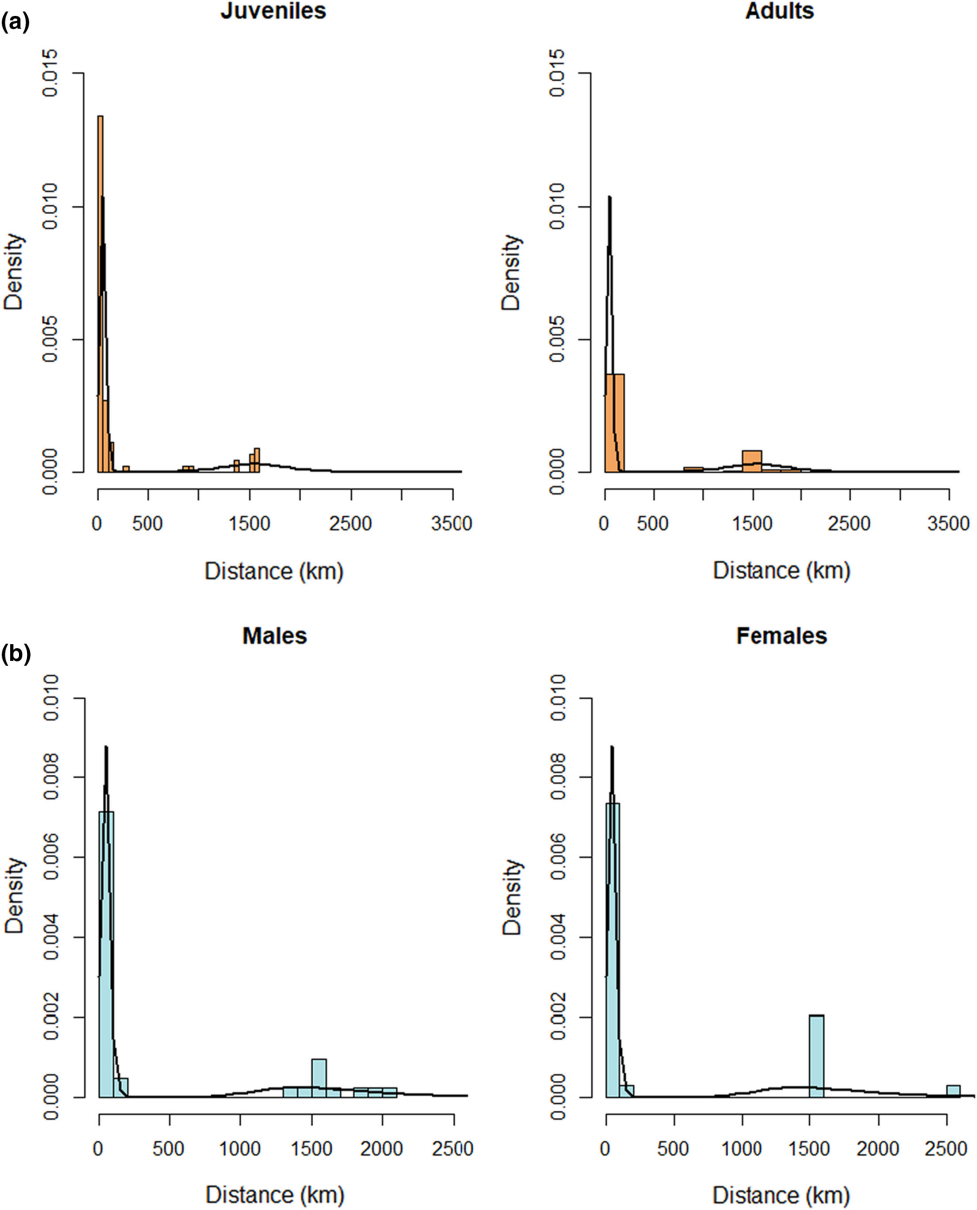
Histograms of the dispersal distance (km) of Glossy Ibis according to age class (juveniles vs. adults) (a) and gender (b) based on fitted two‐component logistic distributions.

Assessment of all two‐component mixtures of continuous distributions indicated that the two‐component logistic and two‐component reverse Gumbel models provided the best fit. Next, we fitted a finite mixture model (two‐component logistic distribution) to dispersal distances without covariates (Table 4). The resulting histogram and two‐density estimates reveal two subgroups: birds that stay in Numidia, and those that disperse long distances (Figure 3a).

**TABLE 4:** Parameters of the fitted two‐component logistic distributions for juvenile and adult Glossy Ibis ringed in Numidian colonies

	Juveniles	Adults
Component 1		
mu (km)	1379.6	1550.9
sigma (km)	210.0	210.0
pi	0.137	0.265
Component 2		
mu (km)	16.4	43.7
sigma (km)	17.1	17.1
pi	0.863	0.735

Finally, we sequentially added age, sex, fledgling mass, wing length, and tarsus length as explanatory variables and compared model fit to the model without covariates. We found that 2Y+ birds display a higher probability than 1Y birds of dispersing further, that is, in and around the Guadalquivir Delta of the Iberian Peninsula. Neither sex (Figure 3b) nor any other biometric measurement appeared to have any influence on the dispersal distance of emigrating Glossy Ibis (ΔAIC < 2).

**FIGURE 4:**
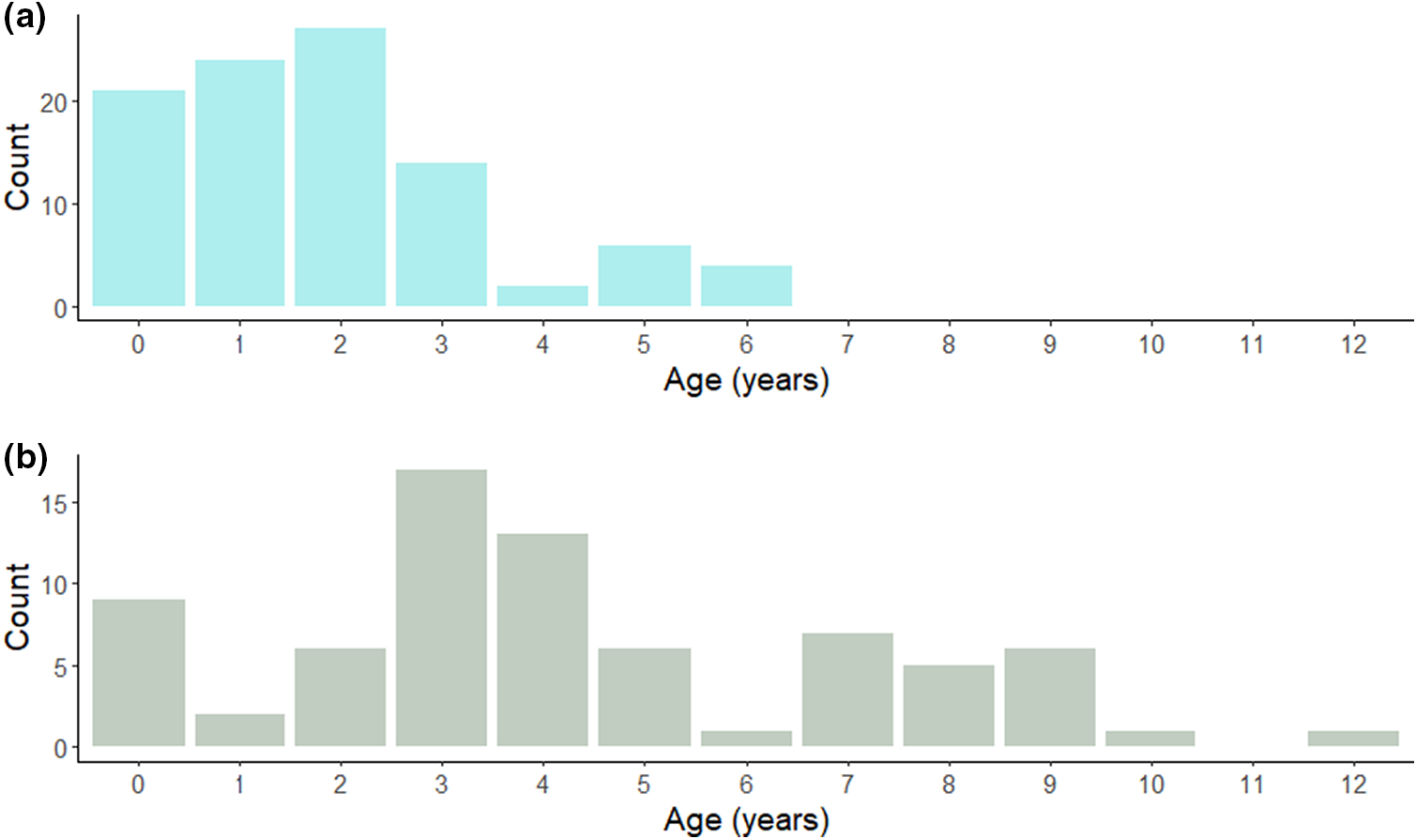
Distribution of the age classes of Glossy Ibis emigrating outside Numidia (a) and immigrating to Numidia from European colonies (b). The age class 0 stands for individuals recorded in their first calendar year (1Y).

The ages of emigrant and immigrant birds imply an age‐related difference in dispersal, with most resightings (73.5%) of Algerian‐born Glossy Ibis outside their natal site involving birds ≤2 years old, with a peak for 2‐year‐old birds (Figure 4a). Median duration of stays abroad for Algerian‐born birds was 12 months (for *N* = 19 birds), with a range spanning 1–68 months, with this latter indicating that at least some Algerian birds settled and likely bred on the Iberian Peninsula. This median duration is clearly a minimum estimate as it represents the interval between two resightings. Immigration to Numidia from European colonies mostly involved older birds, particularly 3‐ and 4‐year‐old birds (Figure 4b). However, some immigrant birds included settlers (up to 12 years old) and probably some transient individuals. The median duration of stay for immigrant birds was 15 months (*N* = 9 birds), with a similarly broad range (0–63 months) to birds emigrating from Algeria.

## 4 DISCUSSION

### 4.1 Recolonization and connectivity between North African and European colonies

Our study shows that following the recolonization of the Maghreb, the population of Glossy Ibis in Algeria has increased substantially and expanded rapidly across the country. Indeed, numbers of new breeding colonies in Numidia have steadily increased (Nedjah et al., 2019). These new colonies have likely benefited from immigration and a high recruitment rate (Santoro et al., 2016). The partial exchange of individuals between North African and European colonies, with some individuals settling and breeding outside their natal site, clearly supports regular gene flow across the vast Mediterranean range of the Glossy Ibis. Despite the relatively high degree of philopatry exhibited by the Numidian population, our results highlight large‐scale connectivity between Algeria and the large Glossy Ibis colonies of southern Europe (mainly in Spain and Portugal, but also in France and Italy) (Champagnon et al., 2019; Curćo Masip & Brugnoli Segura, 2019; Encarnação, 2019; Grussu, 2019; Kazantzidis et al., 2019; Máñez et al., 2019; Volponi, 2019). Thus, our study supports the hypothesis of Hanski and Gilpin (1991) of a Mediterranean‐West African metapopulation of Glossy Ibis.

### 4.2 An Ibero‐Maghrebian flyway?

The Glossy Ibis is well‐known for its long‐distance flight capability, with distances of 3500 km (Schogolev, 1996) and 6000 km (Santoro et al., 2016, 2019) having been documented for juvenile birds. However, apart from the studies of Schogolev (1996) and Santoro et al. (2019), very few studies have explored the regular movements of Glossy Ibis and virtually nothing is known about the dispersal routes of North African birds. Our Glossy Ibis ringing program confirms connectivity between Algerian colonies and others throughout the Mediterranean and West Africa. We consider that the pronounced Ibero‐Maghrebian flyway may be attributable to the distribution of wetlands in the western Mediterranean basin; the Numidian wetland complex is by far the largest in North Africa (Samraoui & Samraoui, 2008), and the Guadalquivir Delta is one of the most important wetlands in Western Europe (Green et al., 2018).

We have thus uncovered a distinctly W/NW‐E/SE‐biased dispersal orientation. Moreover, the high value of the sample mean resultant length (0.8), together with the low measure of dispersion exhibited by the sample circular variance, strongly supports clustering of resighting records around the mean direction. The dominance of this directionality evidences that resightings of Numidian birds on the Iberian Peninsula reflects a genuine link between Algerian and Iberian colonies, similar to the pattern recorded for the Yellow‐legged Gull *Larus michahellis* (Baaloudj et al., 2012). However, whereas dispersal of Yellow‐legged Gull from Algeria was multimodal, with distinct destinations targeting coastal upwelling areas (e.g., Bay of Biscay, Balearic Sea, Galicia, and Gulf of Cadiz) (Baaloudj et al., 2012), Glossy Ibis from Numidia seem to preferentially target the Doñana wetlands in the Guadalquivir Delta. These results are, however, strongly dependent on the spatial heterogeneity of the West Mediterranean landscape and the spatial and temporal heterogeneity of resighting data. Further studies involving different methods and analyses are needed to validate these preliminary results (Schwarz & Bairlein, 2004).

### 4.3 Wind‐assisted dispersal?

A number of studies on birds have reported that long‐distance flights often correlate with favorable winds, based on synchronicity of departure dates and strong tailwinds (Akesson & Hedenström, 2000; Alerstam, 2011; Richardson, 1991). The prevailing winter winds in Numidia are northwesterly, with Mediterranean Mistral and Tramontane winds also being powerfully felt. By contrast, southeasterly winds (Sirocco/Khamsin) sweep across the North African landscape in Spring and Autumn, when the Levanter in the Western Mediterranean is at its strongest. The proportion of Greater Flamingo *Phoenicopterus roseus* that overwinter in Tunisia has been linked to annual variation in the prevalence of favorable winds during the birds' first autumn (Green et al., 1989). Although the distribution of extensive marshlands and the prevailing winds may drive connectivity between the Guadalquivir Delta and Numidia, we cannot rule out the possibility that our results may partially reflect a bias in differential resighting efforts in various European, North African, and West African countries. Thus, further studies are needed to confidently identify the factors driving the Ibero‐Maghrebian connectivity.

### 4.4 Other dispersal routes and flyways

In addition, we have identified some birds taking a more southwesterly dispersal route to other parts of the Maghreb (western Algeria and Morocco) and to West Africa. The lack of records from Tunisia and the relatively large number of sightings from Morocco seems to indicate that Glossy Ibis predominantly move west and then may cross the Gibraltar Strait into Spain and Portugal, supported by one bird being first spotted in Morocco and then in Spain.

Other birds may disperse south along the Atlantic coast to reach West African wetlands, although given that we recorded at least three Algerian‐born birds at El Goléa Oasis where a resident Glossy Ibis colony has been present for the last two decades, it is also possible that Glossy Ibis take a shorter more direct route across the Eastern Sahara (Brown et al., 1982). Emigration to Central Europe and the eastern Mediterranean region from Numidia was less frequent. This eastern route seems less favored by North African birds, perhaps because it involves crossing a broader expanse of the Mediterranean to Italy, with these birds then using that country as a stepping stone to move farther east into Central or southeastern Europe. Eastern Europe was home to huge colonies of Glossy Ibis in the 1970s, before that population suffered a strong and persistent decline (Schogolev, 1996; Zwarts et al., 2009).

Ring recoveries from Ukrainian‐born Glossy Ibis have indicated a clear NE–SW migratory direction, with adults overwintering in the inner Niger Delta but juveniles not straying far from their natal sites (Schogolev, 1996). To reach their wintering grounds in West Africa, adults from these Black Sea colonies must first cross the Mediterranean Sea and then undertake a grueling transit of the Sahara Desert. The directness of this route has been supported by resighting records from Algeria, Tunisia, and Italy, and by the paucity of resightings along the North African Atlantic coast (Santoro et al., 2019; Schogolev, 1996). Glossy Ibis from Caspian Sea colonies follows a more eastern dispersal flyway, with wintering sites in Eastern Africa and the Arabian and Indian peninsulas (Rahmani & Shobrak, 1992; Sapetin, 1978). Exchange does occur between these “eastern” and “western” flyways (Santoro et al., 2019), but warrants further investigation.

### 4.5 Scale‐dependent and age‐related variation in dispersal distances

Most bird species are philopatric, exhibiting relatively low levels of natal and breeding dispersal (Greenwood & Harvey, 1982), with much fewer species undertaking large‐scale movements to find food or nesting sites. Algerian Glossy Ibis seems to encompass both those groups, with a predominantly resident group and a smaller dispersing group. This differential dispersal strategy is similar to a widespread phenomenon termed “partial migration” exhibited by animal populations made up of a mixture of migratory and resident individuals (Chapman et al., 2011; Lundberg, 1988). Nevertheless, dispersal in the Glossy Ibis is scale‐dependent, with even the primarily “resident” group engaging in a certain degree of nomadism at a local scale in response to stressful conditions (e.g., drought or ectoparasitism) (Santoro et al., 2013, this study). Indeed, immigration has been shown to drive rapid growth of new Glossy Ibis colonies at the edge of the species' range (Santoro et al., 2016). This mixed dispersal strategy is likely advantageous for a species inhabiting a spatiotemporally patchy environment (Hamilton & May, 1977; Levin et al., 1984; Picardi et al., 2020; Santoro et al., 2013), and it may well explain the resilience and cosmopolitan range of Glossy Ibis.

Our results indicate that juvenile Glossy Ibis in their first year undertake long‐distance flights and that, overall, the dispersal pattern of the Algerian population is influenced by age. We found that young adult birds (2Y+) were almost twice as likely as 1‐year‐olds to undertake long‐range dispersal. This outcome was somewhat unexpected, as natal dispersal is more typical of immature birds (Greenwood & Harvey, 1982), though some individuals may delay their departure until the breeding season or until they attain maturity. Furthermore, it has been suggested previously that small‐scale avian dispersal may precede large‐scale movements (Altwegg et al., 2000), and prospecting trips are not restricted to immature or subadult birds (Cadiou et al., 1994). Sabbatical (nonbreeding), failed, and successfully breeding birds are inclined to visit other colonies, since there is adaptive value in individuals of all ages gathering information on potential breeding sites in an unpredictable environment (Calabuig et al., 2010; Fijn et al., 2014; Pärt & Doligez, 2003; Reed et al., 1999). It is possible that our results may reflect differential detection probabilities between immature and adult birds or the fact that age at first reproduction in Glossy Ibis may vary between one and 3 years (Burger, 1978; Byrd, 1978; Santoro et al., 2016). Alternatively, our findings may support those of a previous study indicating that long‐range dispersal of 2–4 year‐old Glossy Ibis is driven by kin competition or a failure at the first breeding attempt (Santoro et al., 2016).

Notably, we did not detect any influence of sex or body size on dispersal distances. However, it is possible that the variation in the age of the birds when taking the biometric measurement greatly outweighs possible differences in these variables with respect to dispersal. In most bird species (apart from the Anatidae), dispersal is more prominent among females (Arlt & Pärt, 2008; Clarke et al., 1997; Greenwood, 1980). More extensive sampling may shed further light on the contribution of these and other factors (e.g., breeding success, density, and parasites) on Glossy Ibis dispersal.

### 4.6 How secure is the future for the Glossy Ibis?

It remains unclear what drove extinction of Glossy Ibis colonies across the Mediterranean during the 20th Century. Resolving that question may provide important information on Glossy Ibis dispersal/migration systems (Alerstam, 1990; Remisiewicz, 2002; Sutherland, 1998). Apart from documented cases of anthropogenic persecution or severe habitat alterations, our knowledge of the mechanisms contributing to colony extinction is limited (Beissinger, 2000; Belovsky et al., 1999). A good understanding of the spatiotemporal dynamics of populations is essential to establishing the ecological mechanisms underlying extinction (Owen & Bennett, 2000). The vast current distributional range of the Glossy Ibis may indicate that the factors driving Mediterranean extirpation may not have been localized, but instead exerted an influence over a sizable part of the species' range (Santoro et al., 2013). Rainfall in the Sahel is closely linked to sea surface temperatures, and it has been correlated with precipitation across large parts of Africa and the Mediterranean (Balas et al., 2007; Hulme, 2001; Zwarts et al., 2009). Episodic droughts attributable to altered rainfall patterns, such as the “Great Drought” that devastated the Sahel between 1972 and 1992, may have contributed to the extinction of Mediterranean Glossy Ibis populations during the 20th Century (Zwarts et al., 2009).

Our ringing program has revealed extensive exchange between local colonies and relatively frequent changes in nesting site. Although environmental challenges such as droughts may prompt wider dispersal of wetland birds (Figuerola, 2007; Santoro et al., 2013), it is not always clear why some colonies are abandoned. Glossy Ibis is extremely shy during the breeding period (Erwin, 1989), perhaps owing to acute persecution. This species is subjected to severe poaching in the Sahel (del Hoyo et al., 1992; Zwarts et al., 2009), and we have detected illegal hunting at both foraging and breeding sites in North Africa. We have also recorded at least two cases of breeding site switching due to ectoparasite infestation (water mites) in the previous year. As a result, we suspect that heavy ectoparasite infestation and anthropogenic disturbance (water abstraction and human encroachment) may have been responsible for the low resighting rates of ringed birds from Lake Tonga and Estah, respectively.

A significant cause of concern for Glossy Ibis colonies in Algeria is the potential impact of climate change on dispersal. The physiography of the Mediterranean is highly responsive to climate change (Giogi, 2006), with considerable impacts likely on atmospheric circulation and regional winds (Mistral, Tramontane, Levanter, Libeccio, Sirocco, among others) (Drobinski et al., 2016; Garcia‐Herrera et al., 2014; Ulbrich et al., 2012). Any change in pattern, strength, or frequency of these regional winds may have profound consequences for dispersal of the Mediterranean‐West African Glossy Ibis metapopulation. Other threats have not dissipated since 20th Century population declines, including human encroachment, hunting, habitat fragmentation, pollution, landfills, all of which degrade the Numidian wetland complex that harbors an array of ponds, temporary pools, shallow lakes, and marshes, providing essential breeding and foraging habitats for various species of waders (Samraoui, Nedjah, et al., 2012). Given that dispersal strongly influences biodiversity in all types of ecosystem (Bauer & Hoye, 2014), a clear understanding of animal movement processes can greatly inform management decision‐making and conservation strategies (Driscoll et al., 2014; Picardi et al., 2020).

## AUTHOR CONTRIBUTIONS

**Abdelhakim Bouzid:** Investigation (equal); writing – review and editing (equal). **Abdennour Boucheker:** Investigation (equal); writing – review and editing (equal). **Boudjéma Samraoui:** Conceptualization (equal); formal analysis (equal); methodology (equal); supervision (equal); writing – original draft (equal). **Farrah Samraoui:** Conceptualization (equal); methodology (equal); supervision (equal); writing – review and editing (equal). **Hamed A. El‐Serehy:** Funding acquisition (equal); resources (equal); writing – review and editing (equal). **Riad Nedjah:** Investigation (equal); writing – review and editing (equal).

## FUNDING INFORMATION

King Saud University, Riyadh, Saudi Arabia and its Researchers Supporting Project Number, RSP2023R19.

## Data Availability

Data available on request due to privacy/ethical restrictions. It will also be made available through the Dryad repository after the manuscript acceptance.
